# *Edwardsiella ictaluri* T3SS effector EseN is a phosphothreonine lyase that inactivates ERK1/2, p38, JNK, and PDK1 and modulates cell death in infected macrophages

**DOI:** 10.1128/spectrum.03003-23

**Published:** 2023-10-05

**Authors:** Ranjan Koirala, Chanida Fongsaran, Tanisha Poston, Matthew Rogge, Bryan Rogers, Ronald Thune, Lidiya Dubytska

**Affiliations:** 1 Department of Biological Sciences and Chemistry, Southern University and A & M College, Baton Rouge, Louisiana, USA; 2 Department of Biology, University of Wisconsin-Stevens Point, Stevens Point, Wisconsin, USA; 3 Department of Pathobiological Sciences, Louisiana State University School of Veterinary Medicine, Baton Rouge, Louisiana, USA; Griffith University - Gold Coast Campus, Southport, Gold Coast, Australia

**Keywords:** *Edwardsiella ictaluri*, type III secretion system, T3SS effectors, EseN, apoptosis, phosphothreonine lyase, MAPK, PDK1

## Abstract

EseN is an *Edwardsiella ictaluri* type III secretion system effector with phosphothreonine lyase activity. In this work, we demonstrate that EseN inactivates p38 and c-Jun-N-terminal kinase (JNK) in infected head-kidney-derived macrophages (HKDMs). We have previously reported inactivation of extracellular-regulated kinase 1/2 (ERK1/2). Also, for the first time, we demonstrated that EseN is involved in the inactivation of 3-phosphoinositide-dependent kinase 1 (PDK1), which has not been previously demonstrated for any of the EseN homologs in other species. We also found that EseN significantly affected mRNA expression of *IL-10*, pro-apoptotic *baxa*, and *p53*, but had no significant effect on anti-apoptotic *bcl2* or pro-apoptotic apoptotic peptidase activating factor 1. EseN is also involved in the inhibition of caspase-8 and caspase-3/7 but does not affect caspase-9 activity. Repression of apoptosis was further confirmed with flow cytometry using Alexa Fluor 647-labeled annexin V and propidium iodide. In addition, we found that the *E. ictaluri* T3SS is essential for the inhibition of IL-1β maturation, but EseN is not involved in this process. EseN did not affect cell pyroptosis, as indicated by the lack of EseN impact on the release of lactate dehydrogenase from infected HKDM. The transmission electron microscopy data also indicate that HKDM infected with WT or an *eseN* mutant died by apoptosis, while HKDM infected with the T3SS mutant more likely died by pyroptosis. Collectively, our results indicate that *E. ictaluri* EseN is involved in inactivation of ERK1/2, p38, JNK, and PDK1 signaling pathways that lead to modulation of cell death among infected HKDMs.

**IMPORTANCE:**

This work has global significance in the catfish industry, which provides food for increasing global populations. *E. ictaluri* is a leading cause of disease loss, and EseN is an important player in *E. ictaluri* virulence. The *E. ictaluri* T3SS effector EseN plays an essential role in establishing infection, but the specific role EseN plays is not well characterized. EseN belongs to a family of phosphothreonine lyase effectors that specifically target host mitogen activated protein kinase (MAPK) pathways important in regulating host responses to infection. No phosphothreonine lyase equivalents are known in eukaryotes, making this family of effectors an attractive target for indirect narrow-spectrum antibiotics. Targeting of major vault protein and PDK1 kinase by EseN has not been reported in EseN homologs in other pathogens and may indicate unique functions of *E. ictaluri* EseN. EseN targeting of PDK1 is particularly interesting in that it is linked to an extraordinarily diverse group of cellular functions.

## INTRODUCTION

Enteric septicemia of catfish is an economically important disease of farmed-raised channel catfish, *Ictalurus punctatus*, and is caused by the Gram-negative bacterial pathogen *Edwardsiella ictaluri*. In many Gram-negative pathogens, type III secretion systems (T3SS) are an important component of virulence (1). Previously, we reported that a T3SS is essential for *E. ictaluri* virulence and intracellular replication (2) and plays an important role in programmed cell death of host cells (3). T3SS serve to deliver effector proteins into eukaryotic cells (1). The T3SS effectors are a large and diverse group of virulence proteins that mimic eukaryotic proteins in structure and function and target a variety of eukaryotic physiological functions (4).

Many of these effector proteins are enzymes that chemically alter host proteins to interrupt or rewire host signaling pathways, thereby promoting bacterial survival and replication (5 – 7). The modifications to the signaling pathways by a subset of pathogen effectors differ from host modifications in their irreversibility (8 – 12). Phosphothreonine lyases are one example of such an enzyme that removes phosphate without hydrolysis and in a manner that is not reversible (8, 12, 13). For the MAPK signaling systems, such chemical transitions have significant impact on innate immune signaling. When MAPKs are involved in combating bacterial infections and activating the innate immune response, signal transfer and pathway activation are based on rapidly reversing cascades of phosphorylation and dephosphorylation. Irreversible dephosphorylation can prevent all further signal transmissions regardless of changes in conditions and leaves cells vulnerable or defenseless to infection (9, 11, 14).

Recently, the substrate requirements for several phospholyases have been reported (15). For example, *Shigella flexneri* T3SS effector, OspF, can interfere with many host processes simultaneously, while *Salmonella enterica* SpvC is more specific for the MAPK activation loop. Each phospholyase may have its own preferred substrate and/or modify an array of substrates in host cells (15). Earlier, we demonstrated that *E. ictaluri* effector protein EseN belongs to a family of T3SS effector proteins with phosphothreonine lyase activity that inactivates extracellular-regulated kinase 1/2 (ERK1/2) (16). We have also demonstrated that EseN is involved in inhibition of the M1 phenotype of head-kidney-derived macrophages (HKDMs) in response to *E. ictaluri* infection (17) and is involved in modulation of pathways that control the immune response to infection and expression of transcription factors NF-κβ (c-*rel* and *relB*), *creb3L4*, *socs6*, and *foxo3a* (17). We have also demonstrated that *E. ictaluri* effector EseN interacts with the major vault protein (MVP) that can serve as a signaling scaffold for selecting specific MAPKs for inactivation during *E. ictaluri* infection (16, 18).

In this work, we demonstrated that *E. ictaluri* EseN, similar to its homologs *Shigella* OspF and *Salmonella* SpvC, can inactivate p38 and c-Jun-N-terminal kinase (JNK) MAPKs. Moreover, for the first time, we demonstrated that EseN is involved in inactivation of 3-phosphoinositide-dependent kinase 1 (PDK1) in phorbol-12-myristate 13-acetate (PMA) pretreated HKDM, a feature that has not been demonstrated in other EseN homologs. PDK1 is a serine/threonine-protein kinase that belongs to the AGC kinase family and plays a central role in regulating cell growth, survival, and proliferation (19). Inactivation of PDK1 within infected HKDM may demonstrate a unique mechanism by which EseN effects host cell responses. To further develop the model for *E. ictaluri* pathogenesis, we also investigated the involvement of EseN in regulation of programmed cell death by direct measurement of apoptotic caspase-3/7, caspase-8, and caspase-9 activities and quantifying apoptotic cells by flow cytometry using Alexa Fluor 647-labeled annexin V (AnnV) and propidium iodide (PrI). We also analyzed mRNA expression of several genes that play important roles in activation or inhibition of apoptosis, including apoptotic peptidase activating factor 1 (*apaf1*) (20), *p53* (21), and *baxa* (22). Becasue IL-10 can correlate with apoptosis (23), *IL-10* mRNA expression was also analysed. To examine the involvment of EseN in pyroptosis, we investigated IL-1β maturation and measured cytotoxicity through the detection of lactate dehydrogenase (LDH) release by infected HKDM.

## RESULTS

### EseN dephosphorylates pp38 and pJNK *ex vivo*

**Fig 1:**
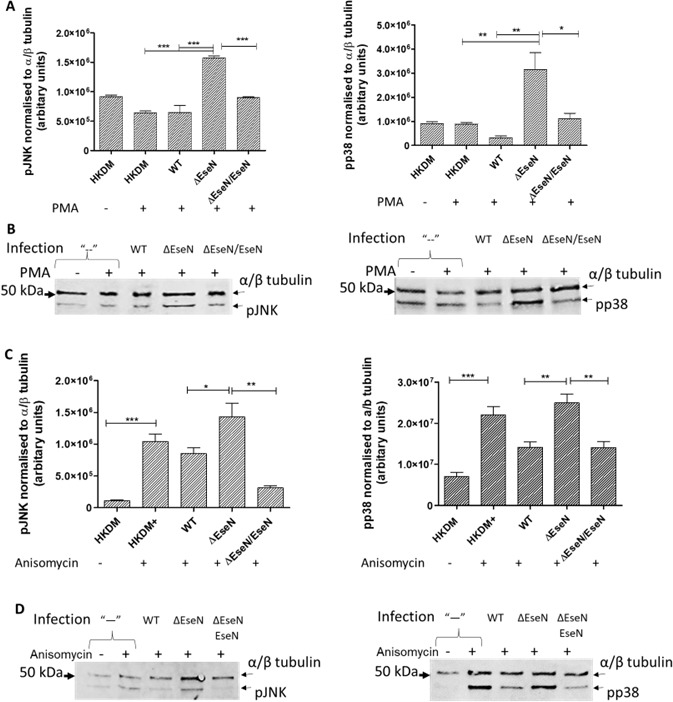
Western blot results showing inactivation of JNK and p38 *ex vivo*. (**A and C**) Levels of activation by phosphorylation of p38 and JNK detected in panels B and D, except quantified using a ChemiDoc MP imaging System (Bio-Rad) with bands normalized to α/β-tubulin. Comparison between groups was based on one-way analysis of variance with Tukey’s post hoc procedure for comparison of group means. Mean ± SD, *N* = 4–5, depending on treatment. Panels B and D show representative experiments that detect phosphorylated JNK and p38, *ex vivo* in uninfected channel catfish head-kidney-derived macrophages (HKDMs) infected with wild-type (WT) *Edwardsiella ictaluri*, mutant EseN (*ΔeseN*), or the *ΔeseN* complemented strain (*ΔeseN/eseN*) following stimulation for 15 min with phorbol-12-myristate 13-acetate (PMA) (**B**) or anisomycin (**D**). Both WT and the *ΔeseN/eseN* complemented strain inactivated p38 and JNK in HKDM stimulated with PMA (**A and B**) and anisomycin (**C and D**). *P < 0.05; **P < 0.01; ***P < 0.001*.*

PMA does not significantly stimulate activation of p38 and JNK in uninfected HKDM (Fig. 1A and B; compare HKDM+ to HKDM−). In contrast, anisomycin, which is a specific activator of p38 and JNK (24), significantly stimulates activation of p38 and JNK in uninfected HKDM (Fig. 1C and D; compare HKDM + to HKDM−). Levels of pp38 and pJNK in PMA or anisomycin-stimulated HKDM were significantly reduced when HKDMs were infected with wild type (WT) and *ΔeseN* complement (ΔeseN/eseN) compared to HKDM infected with *ΔeseN* (Fig. 1). These results indicate that EseN inactivates pp38 and pJNK during *E. ictaluri* infection.

### EseN interacts with PDK1 in Y2H assay

Previously, we described the cloning of an EseN “bait” into pDEST32 and the making of a HDKM complementary DNA (cDNA) library in pDONR22 to act as “prey” in this Y2H system (16). Successful association of proteins in this assay is indicated by the expression of URA3 and HIS3 (two of three reporters) and the ability of the colony to grow without supplemented uracil and histidine, respectively. In addition to MVP (16), we recovered one colony that grew poorly without the uracil but grew well on medium lacking histidine in the Y2H assay.

This clone was then re-screened on medium lacking histidine with different concentrations of competitive inhibitor of the reporter HIS3 gene: 3-amino-1,2,4-triazole (3-AT). The previously described pDONR22-MVP was used as a positive control for interaction, while an empty pDONR22 was a negative control. The clone grew on media lacking histidine and on media lacking histidine and containing 10- and 25-mM 3-AT but not 50-mM 3-AT, indicating a moderate interaction between EseN and the cloned protein. This clone was also URA3-positive and sensitive to 0.2% 5-fluoroorotic acid, which is converted by URA3 to fluorodeoxyuridine, a toxic analog of uracil.

**Fig 2:**
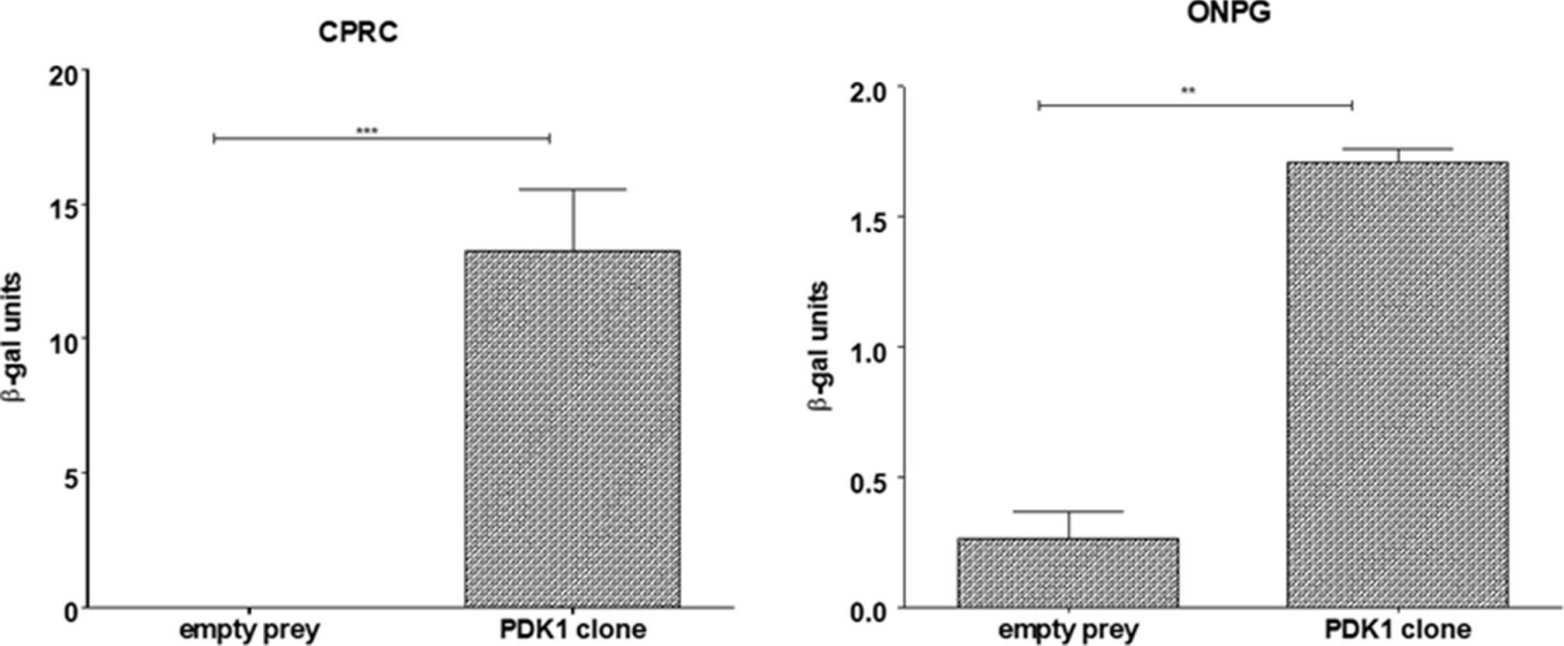
Quantitative assays for β-galactosidase (β-gal) activity for PDK1 clone in liquid culture using chlorophenol red-β-D-galactopyranoside (CPRG) or o-nitrophenyl-β-D-galactopyranoside (ONPG) as substrate. As a substrate, CPRG is more sensitive and the reaction time is faster than ONPG, but the strength of the reaction indicates the relative strength of the bait/prey interaction in both cases. Statistical analysis was conducted using *t*-test. Data are mean ± SD of three replicates. **P < 0.01, ***P < 0.001.

The pDONR22 from this clone was then purifide and sequenced. Based on a BLAST of the insert sequence, pDONR22 contained a 95% *pdk1* cDNA. To confirm interaction, pDEST23-PDK1 and pDONR22-EseN were retransformed into the host, *Saccharomyces cerevisiae* strain MaV203. Activation of the reporter genes following transformation and quantitation of beta-galactosidase activity using o-nitrophenyl-β-D-galactopyranoside and chlorophenol red-β-D-galactopyranoside once again confirmed the interaction of EseN and PDK1 (Fig. 2).

### EseN dephosphorylates pPDK1 *ex vivo*

**Fig 3:**
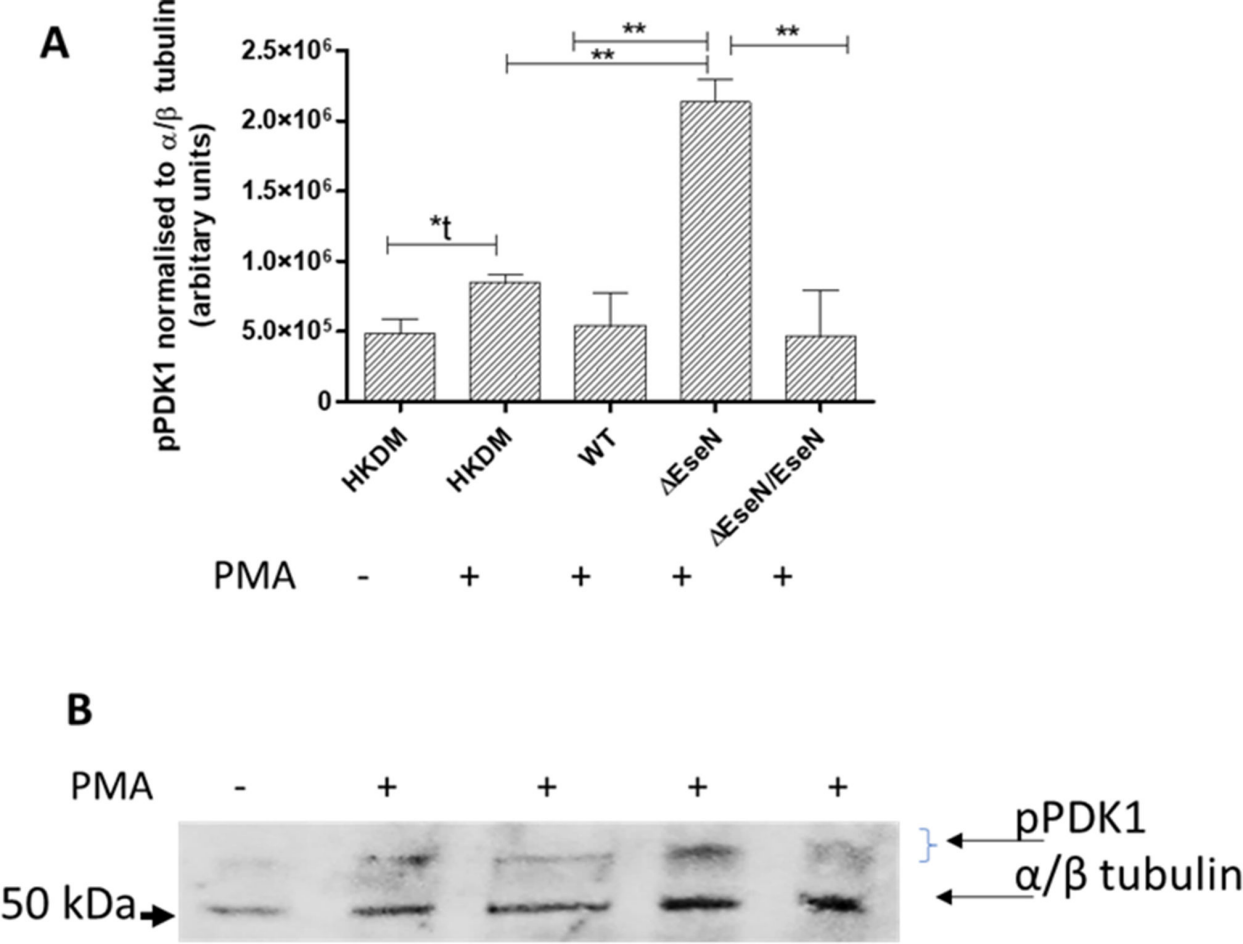
Western blot results of PDK1 inactivation. (**A**) Levels of activated pPDK1 as detected in panel **B**, quantified using a ChemiDoc MP imaging System (Bio-Rad) with bands normalized to α/β-tubulin. Comparison between groups was based on one-way analysis of variance with Tukey’s post hoc procedure for comparison of group means. Mean ± SD, *N* = 4–5, depending on treatment. (**B**) Result of a representative experiment detecting phosphorylated PDK1, *ex vivo* in uninfected channel catfish head-kidney-derived macrophages (HKDMs) infected for 15 min with wild-type (WT) *Edwardsiella ictaluri*, mutant *eseN* (*ΔeseN*), or the *ΔeseN* complemented strain (*ΔeseN/eseN*) following stimulation with phorbol-12-myristate 13-acetate (PMA). Both WT and the *ΔeseN/eseN* complemented strain inactivate PDK1 in HKDM stimulated with PMA. *P ≤ 0.05, **P ≤ 0.01.

To test if EseN is involved in inactivation of PDK1, we performed Western blot analyses of uninfected HKDM and HKDM infected with *E. ictaluri* WT, *∆eseN*, and *∆eseN*/*eseN*. PMA significantly (by *t*-test) stimulated PDK1 activation in uninfected HKDMs (Fig. 3A; compare HKDM+ to HKDM−). Dephosphorylated PDK1 was detected in the PMA-treated HKDM (Fig. 3) but was not detected in anisomycin-treated HKDM or untreated HKDMs. This observation is consistent with the fact that PMA can activate phosphoinositide 3-kinase (PI3K) (25) and lead to PDK1 activation, while anisomycin is a specific activator of p38 and JNK and does not affect PDK1 activation. Importantly, levels of pPDK1 in PMA-stimulated HKDM infected with WT and *ΔeseN*/*eseN* were significantly reduced compared to cells infected with *ΔeseN* (Fig. 3A and B). These results indicate that EseN is involved in pPDK1 dephosphorylation during *E. ictaluri* infection.

### EseN modulates expression of genes involved in cell death

**Fig 4:**
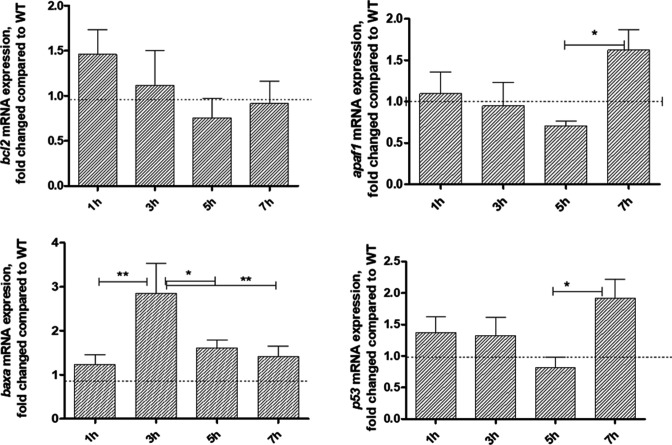
Fold changes of mRNAs expressed from HKDM infected with *E. ictaluri ∆eseN* compared to HKDM infected with *E. ictaluri* WT. Expression of mRNAs was measured by RT-qPCR at different time points post-infection. Data were collected and analyzed by Roche LightCycler 96 qPCR and software using relative expression method. *CanX* was used as the reference gene. Bars indicate fold changes in mRNA expression of HKDM infected with *∆eseN* compared to WT. Fold changes were calculated after all data were normalized to the reference gene by dividing the mRNA expression in HKDM infected with *∆eseN* by the number of mRNA expression in HKDM infected with WT. Results are presented as means and standard errors of the means and are combined data from four to five identical experiments, with three replications per treatment per experiment. *P ≤ 0.05, **P ≤ 0.01.

Because MAPKs and PDK1-AKT pathways are involved in regulation of cell survival and death, we studied the role of EseN in those processes. We analyzed mRNA expression of several genes that play important roles in activation or inhibition of apoptosis (Fig. 4), including anti-apoptotic *bcl2* (26), *apaf1*, a key indicator of apoptosis in the intrinsic pathway (20), pro-apoptotic gene *baxa* (22), and *p53*, which plays a role in controlling cell division and cell death (21). Levels of mRNAs were measured at 1, 3, 5, and 7 h post-infection (PI) in uninfected HKDMs and HKDMs infected with WT and *E. ictaluri ΔeseN* by reverse transcription quantitative real-time PCR (RT-qPCR).

We did not detect significant differences in mRNA expression of anti-apoptotic *bcl2* among HKDMs infected with any strains or at any time of infection (Fig. 4, top left). Results with *apaf1* were similar to *bcl2* except that the *apaf1* mRNA expression ratio in HKDMs infected with *ΔeseN* was significantly higher at 7 h compared to 5 h PI (Fig. 4, top right). In contrast, EseN significantly affected mRNA expression compared to the two other tested pro-apoptotic genes. The mRNA levels were significantly upregulated in HKDMs infected with *∆eseN* compared to WT at 3 h of infection for *baxa* and at 7 h of infection for *p53* (Fig. 4, bottom left and bottom right).

**Fig 5:**
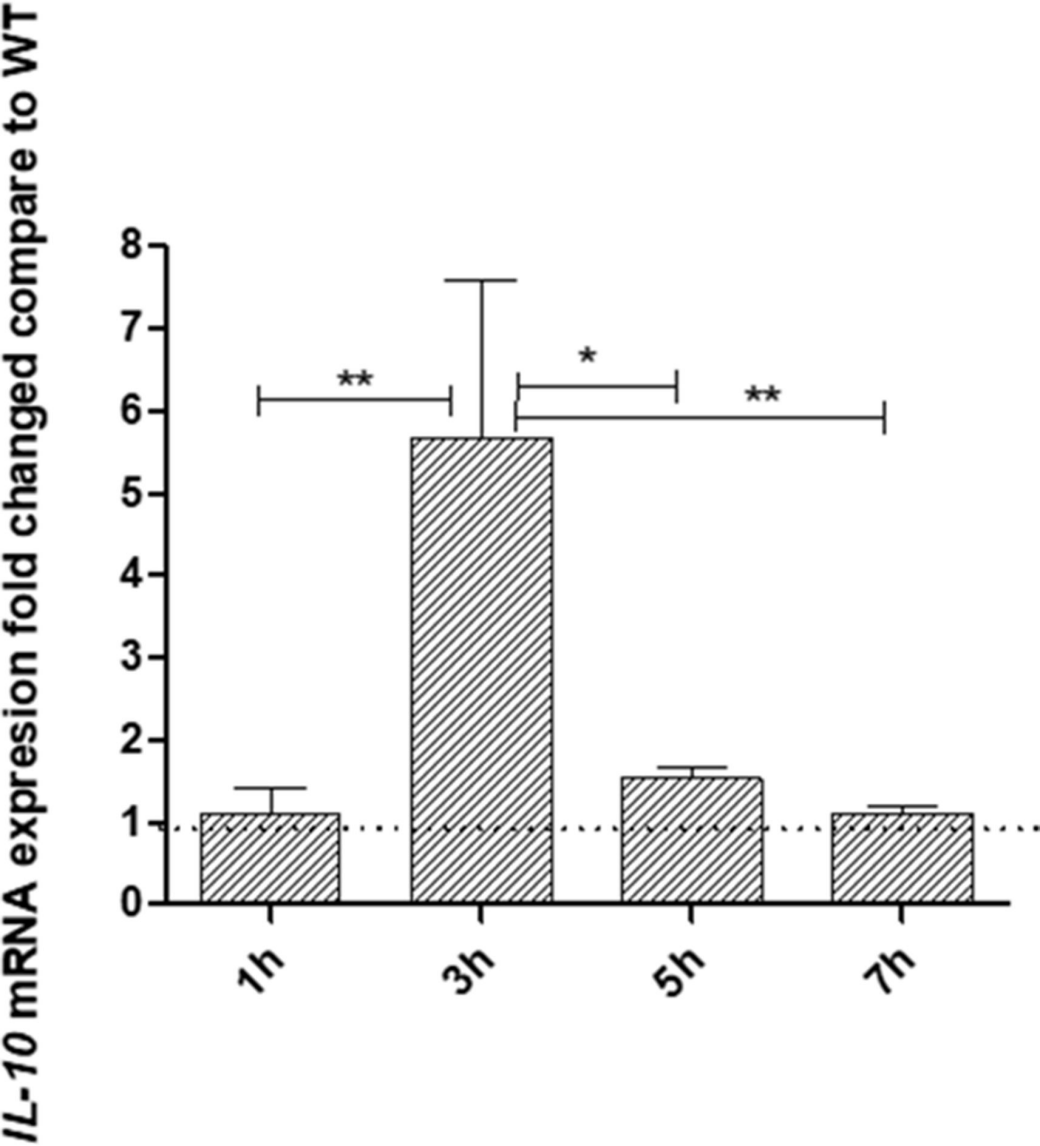
Fold changes of *IL-10* mRNA expressed from HKDM infected with *E. ictaluri ∆eseN* compared to HKDM infected with *E. ictaluri* WT. expression of mRNAs was measured by RT-qPCR at different time points post-infection. Data were collected and analyzed by Roche LightCycler 96 qPCR and software using relative expression method. *CanX* was used as the reference gene. Bars indicate fold changes in mRNA expression of HKDM infected with *∆eseN* compared to WT. Fold changes were calculated after all data were normalized to the reference gene and uninfected HKDMs by dividing the mRNA expression in HKDM infected with *∆eseN* by the number of mRNA expression in HKDM infected with WT. Results are presented as means and standard errors of the means and are combined data from four to five identical experiments, with three replications per treatment per experiment. *P ≤ 0.05; **P ≤ 0.01.

It was reported that expression of lL-10 correlates with apoptosis (23) in infected HKDM. Therefore, we investigated *IL-10* mRNA expression in HKDMs infected with WT and *∆eseN* using uninfected HKDMs as a negative control. Infection with *∆eseN* induced *IL-10* production at 3 h PI compared to infection with WT at 3 h PI (Fig. 5). During 1, 5, and 7 h PI, no significant differences in *IL-10* mRNA expression were detected between HKDM infected with WT or *∆eseN*. Collectively, these data indicate that during *E. ictaluri* infection, EseN is required for inhibition of *baxA*, *p53*, and *IL-10* mRNA expressions that inhibit apoptosis in infected HKDMs.

### Relationship between EseN and HKDM apoptosis

**Fig 6:**
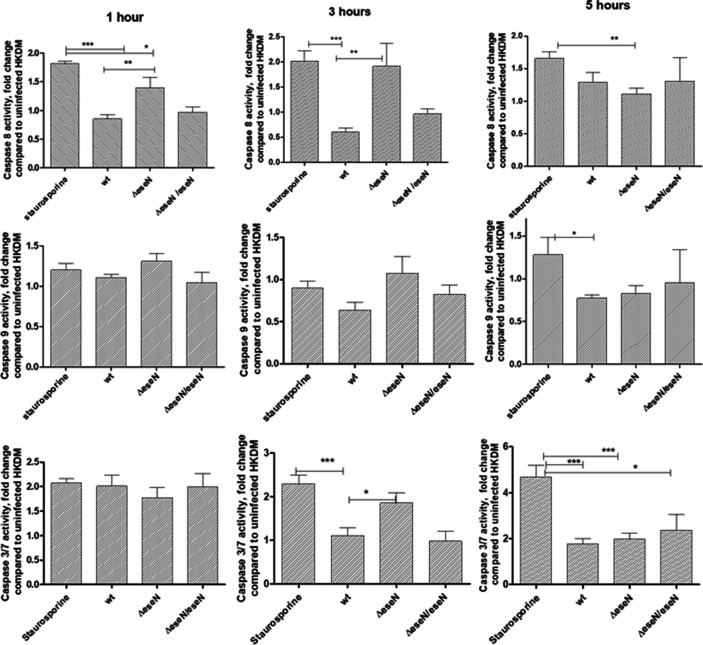
Caspase activities in HKDM cells infected with *E. ictaluri* WT and *∆eseN*. Staurosporine-treated HKDM was used as a positive control. Cultures were harvested at 1, 3, and 5 h post-infection. Values are expressed as means ± standard deviation of four independent experiments. Asterisks indicate significant differences following one-way analysis of variance with Tukey’s post hoc test to compare the mean of each treatment with every other treatment. Asterisks indicate significant difference between treatments. *P < 0.05, **P < 0.01, ***P < 0.001.

Caspase-8 activity in *ΔeseN*-infected HKDM was elevated at 1 and 3 h post-infection (Fig. 6) and did not differ at 7 h PI (Fig. 6, top row). Caspase-9 activity in WT-infected cells was lower than in *∆eseN*-infected cells at 3 h PI only, but this difference was not significant (Fig. 6, middle row). Caspase-3/7 activity increased significantly in the *∆eseN*-infected HKDM compared to WT-infected cells after 3 h PI but then declined to a WT level at 5 h PI. These data indicate that EseN is involved in inhibition of early apoptotic caspase-8 and suicide caspase-3/7 in infected HKDMs.

**Fig 7:**
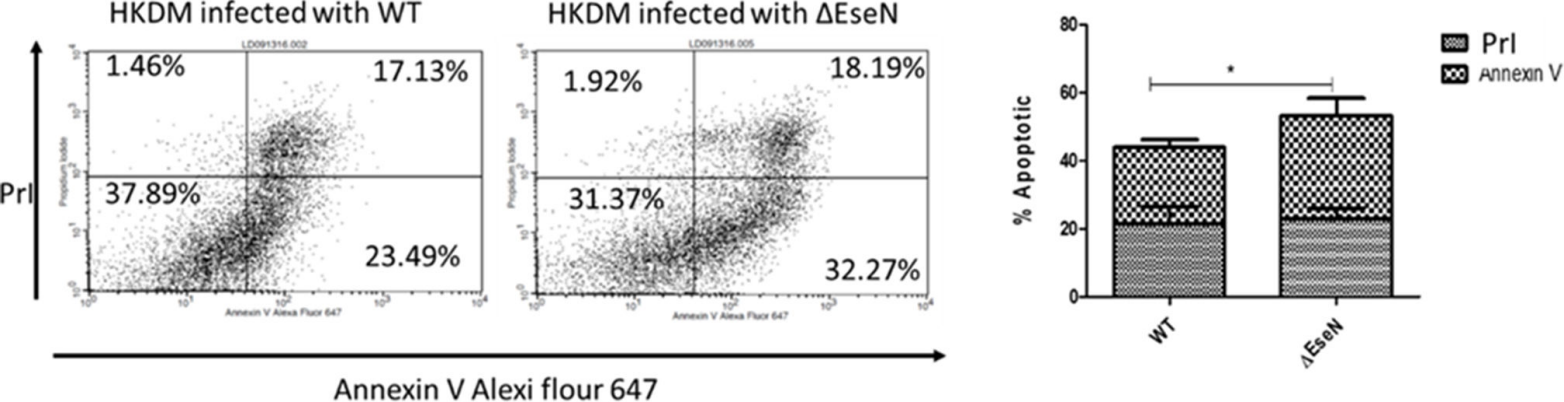
Assessment of HKDM by flow cytometry after annexin V/propidium iodide labeling. (**A**) Results are presented for one of three representative experiments. (Lower left) The percentage of cells that were viable (AnnV−/PrI−); (lower right) early apoptotic (AnnV+/PrI−); (upper right) late apoptotic (Ann+/PrI+). (**B**) Graph of the mean percentages of early and late apoptotic cells for the three experiments shows that the apoptotic HKDM infected with ∆EseN mutant was significantly greater than the WT. *P ≤ 0.05.

The relationship between EseN and macrophage apoptosis was further investigated by assessing the surface expression of phosphatidylserine using Alexa Fluor 647-labeled AnnV in conjunction with PrI after 5 h PI (Fig. 7). Early apoptotic cells bind annexin V, a Ca^2+^-dependent phospholipid-binding protein with a high affinity for externalized phosphatidylserine. Propidium iodide stains double-stranded DNA in cells with damaged cell membranes but fails to penetrate and stain cells with intact membranes. As shown in Fig. 7, 37.89% of WT-infected HKDM cells and 31.37% of *∆eseN*-infected HKDM were viable (AnnV−/PrI−). In addition, 23.49% of WT-infected cells were early apoptotic (AnnV+/PrI−), and 17.13% were late apoptotic (Ann+/PrI+), for a total of 40.63% positive for apoptosis. In contrast, infections with *∆eseN* resulted in a significant increase in the level of early apoptotic cells at 32.27% but no significant increase in late apoptotic cells at 18.19% of total cells, for a total of 50.46% positive for apoptosis.

### *IL-1β* activation during *E. ictaluri* infection of HKDM

**Fig 8:**
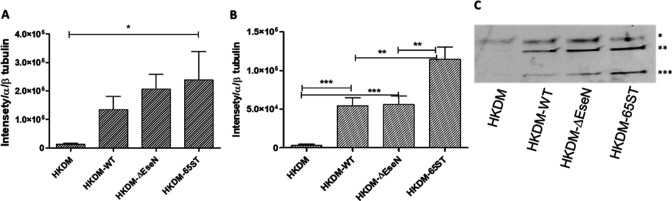
Inhibition of IL-1β maturation in HKDMs infected with *E. ictaluri*. (**A and B**) Graphical representation of pro-IL-1β (**A**) and IL-1β (**B**) expression in HKDM infected with either WT *E. ictaluri*, *∆eseN*, or the T3SS mutant 65st based on the band intensities in Western blots from four independent experiments. Values are expressed as means ± standard deviation of four independent experiments. Asterisks indicate significant differences following one-way analysis of variance with Tukey’s post hoc test to compare the mean of each treatment with every other treatment. Asterisks indicate significant difference between treatments. *P < 0.05, **P < 0.01, ***P < 0.001. (**C**) Representative Western blots that detected pro-IL-1β and IL-1β *ex vivo* in uninfected HKDMs, HKDM infected with wild-type (WT) *Edwardsiella ictaluri*, mutant EseN (*ΔeseN*), and 65ST. *, α/β-tubulin; **, pro-IL-1β; ***, matured IL-1β.

To investigate if T3SS effector EseN can trigger macrophage cell death by inflammation, we evaluated IL-1β release and maturation. Western blot analysis showed that *E. ictaluri* infection activates IL-1β expression in infected HKDM (Fig. 8A). Differences between uninfected HKDM and HKDM infected with *∆eseN* or T3SS mutant 65ST (2) were significant (*P* < 0.01) by non-parametric Kruskal-Wallis test and not significant by one-way analysis of variance (ANOVA) (Fig. 8A). Maturation of pro-IL-1β to IL-1β was detected in all infected HKDM independent of the bacterial strain (Fig. 8B and C). However, we did not detect any differences in IL-1β maturation between HKDM infected with WT and HKDM infected with *∆eseN* (Fig. 8B). Maturation of pro-IL-1β to IL-1β in HKDM infected with 65ST was significantly higher than that in HKDM infected with WT or *∆eseN*, suggesting that other T3SS effectors are involved in inhibition of IL-1β maturation.

### Cellular damage in macrophages caused by invasion of *E. ictaluri*

**Fig 9:**
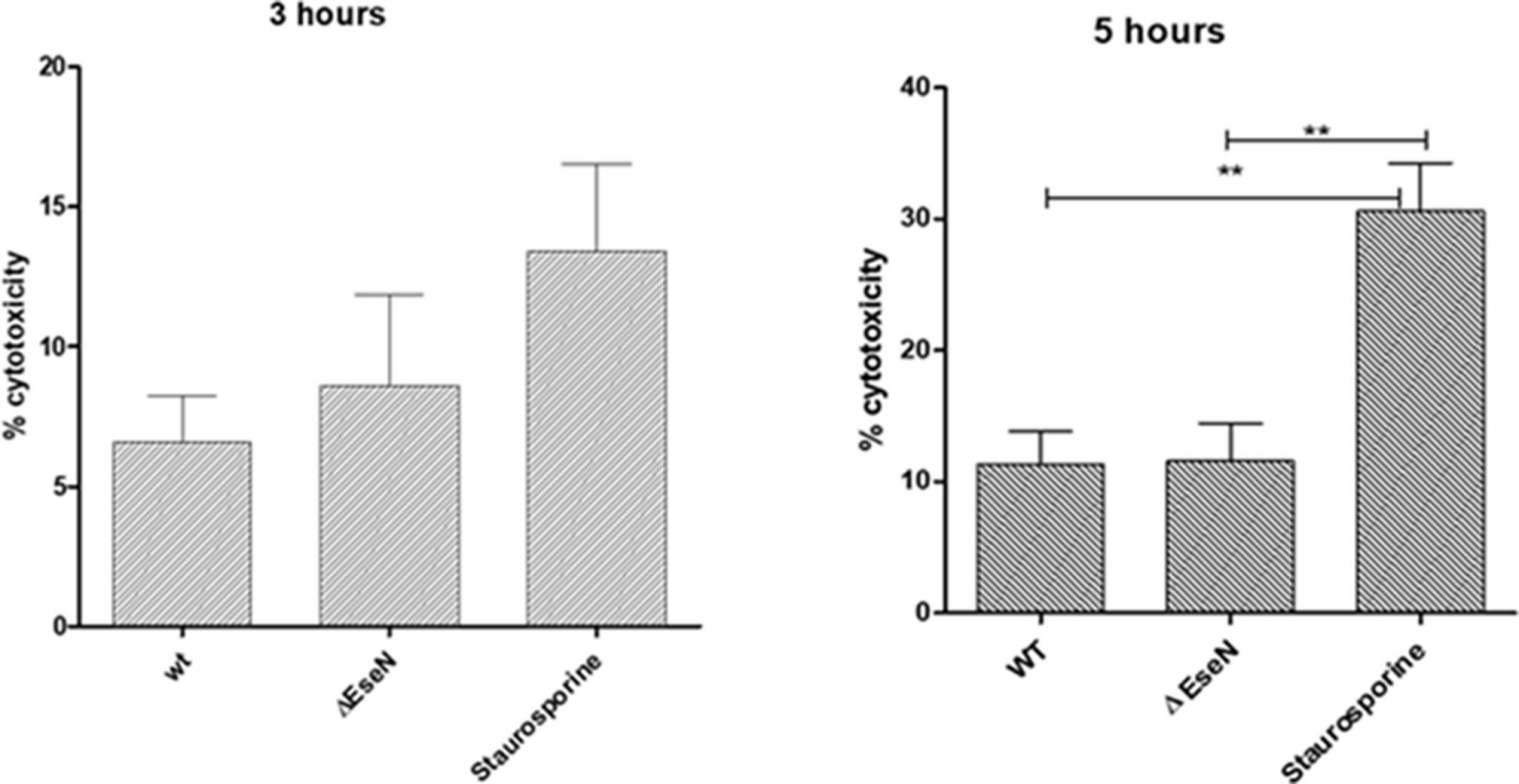
Release of LDH by HKDM infected with *E. ictaluri* WT and *∆eseN*. Cytotoxicty was based on release of LDH and detected using the CytoTox-ONE homogeneous cytotoxicity assay as described in Materials and Methods. Staurosporine-treated HKDM were used as a positive control. One-way analysis of variance with Tukey’s post hoc test was used to compare the mean of each treatment with every other treatment. Asterisks indicate significant difference between treatments (*N* = 3). **P < 0.01.

**Fig 10:**
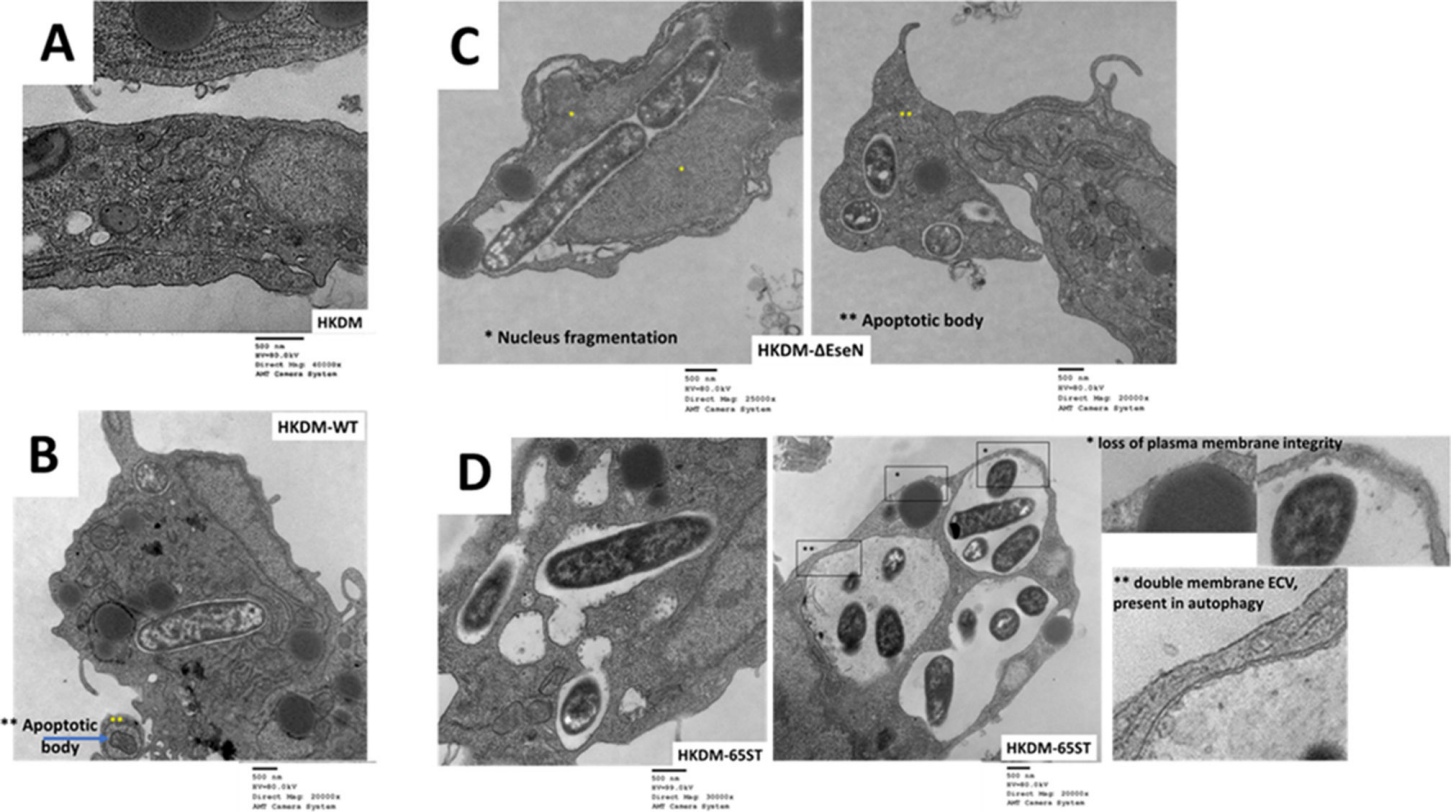
Transmission electron microscopy images of HKDM infected with different strains of *E. ictaluri*. (**A**) Uninfected HKDM showing normal structure and intact plasma membrane. (**B**) HKDM infected with WT showing intracellular *E. ictaluri* and apoptotic bodies (**). (**C**) HKDM infected with ∆*eseN* showing nuclear fragmentation (*) and apoptotic bodies (**). (**D**) HKDM infected with 65ST, showing large vacuoles containing *E. ictaluri*, double membranes (**, inset) typical of autophagy, and loss of plasma membrane integrity (*, inset). Infected cells were harvested 5 h PI, fixed, and prepared for TEM.

To investigate if EseN plays any role in *E. ictaluri* cytotoxicity, we assessed membrane damage in HKDMs infected with WT or *∆eseN* by measuring LDH release (Fig. 9) and examined cells with transmission electron microscopy (TEM) (Fig. 10C and D). There was no difference in cytotoxicity as shown by LDH release (Fig. 9) between HKDM infected with WT or *∆eseN*. We also did not see membrane damage with EM (Fig. 10C and D).

### Transmission electron microscopy of HKDM infected with *E. ictaluri* strains

TEM was used to visualize HKDM cell death in HKDM infected with WT, *∆eseN*, and 65ST. Uninfected HKDM exhibited intact membrane and surface projections characteristic of healthy cells (Fig. 10A). Infection of HKDM with WT led to the formation of apoptotic bodies and nuclear shrinkage, but HKDMs maintained intact membranes (Fig. 10B). HKDM infected with *∆eseN* demonstrated similar characteristics to HKDM infected with WT (Fig. 10C), except the number of cells forming apoptotic bodies was greater. Furthermore, in *∆eseN*-infected HKDM, we detected nuclear fragmentation as well as apoptotic body formation. The HKDM infected with 65ST significantly differed from HKDM infected with WT and *∆eseN*. Infection by 65ST caused formation of large vacuoles, many of which were surrounded by a double membrane which can indicate autophagy (Fig. 10D). Many cells also lost plasma membrane integrity, which correlates with our cytotoxicity study that was previously reported (3).

## DISCUSSION

The *E. ictaluri* T3SS effector EseN belongs to a family of phosphothreonine lyases. EseN has 63% and 71% AA identity to the known T3SS effectors OspF in *Shigella* spp. and SpvC in *Salmonella* spp., respectively (27). Activation of MAPKs during WT infection leads to transcriptional reprogramming and induction of an innate immune response (28 – 31). However, subsequent dephosphorylation of pERK1/2 (16), pp38, and pJNK by EseN (Fig. 1) results in production of inactive ERK1/2, p38, and JNK, which downregulates the host inflammatory response (32, 33) and enhances proliferation of *E. ictaluri* (16). This is further supported by the reduction of the quantity of *E. ictaluri* in head-kidney following infection with the *ΔeseN* strain compared to the WT, as well as by a reduction in mortality (16). Interaction of EseN with the scaffold protein MVP (16) could regulate selection of MAPKs for dephosphorylation during the infection. Expression of EseN is enhanced during *E. ictaluri* invasion (34) of HDKM, which can lead to decreased phosphorylation levels of ERK1/2 (16), JNK, and p38. This MAPK inactivation results in the downregulation of the host immune response (17) and could at least be partially responsible for the death of infected HKDM.

Interestingly, EseN is also involved in the dephosphorylation of PDK1 (Fig. 3). PDK1 activates downstream kinases, like Akt (35, 36), and has been shown to regulate protein synthesis, cell survival/death, glucose metabolism, and cell adhesion and migration. Multiple serine sites are phosphorylated on PDK1, and it has been demonstrated that serine 241 phosphorylation is required for PDK1 activity (35, 36). Tyrosine (Tyr-373/Tyr-376) phosphorylation may also regulate PDK1 activity (35, 36). Unfortunately, specific antibody to Tyr-373/Tyr-376 was not available for our study. Importantly, inactivation of PDK1 has not been demonstrated for any EseN homologs, indicating a possible unique function of this T3SS effector. Nevertheless, it was shown that *S. flexneri* effector OspF alters the phosphorylation of several hundred proteins, thereby demonstrating its broad impact during infection (37).

It was previously reported that MAPKs can be involved in regulation of programmed cell death (38 – 40). Cross-talk signaling between JNK, ERK1/2, and p38 MAPKs are important regulatory mechanisms in stress responses (41, 42). These kinases function in a cell context-specific and cell type-specific manner and integrate signals at different points through both transcriptional and post-translational mechanisms, which can result in caspase activation (38).

A diverse set of JNK and p38 MAPK substrates and transcription factors that are regulated by those MAPKs have been identified and validated (43 – 45). One of the best known transcription factors regulated by the JNK and p38 MAPK cascades in apoptosis is p53 tumor suppressor protein. In stressed cells, JNK-mediated phosphorylation can stabilize and activate p53 and thus promote programmed cell death (46) in combination with other proteins. It was reported that p53-p73 dimerization is critical in the induction of apoptotic cell death, particularly as part of the JNK-mediated cell stress response. The p53-p73 dimer facilitates the expression of several pro-apoptotic target genes, such as *puma* and *bax* (47). Our data indicate that EseN was involved in regulation of *p53* mRNA expression in a time-dependent manner and was upregulated only at 7 h PI (Fig. 4). EseN also inhibits pro-apoptotic *bax* mRNA expression at 3 h PI but has no effect on anti-apoptotic *bcl-*2 mRNA expression. Yue and López reported that *bax* and *bcl-2* are under the control of JNK and/or p38 MAPK cascades at a transcriptional and/or post-transcriptional level (38).

Previously, we reported that *E. ictaluri* T3SS effector EseN is involved in regulation of *foxo3a* mRNA expression in infected HKDM (17). FOXO3a mediates multiple physiological and pathological processes by inducing transcription of target genes involved in apoptosis (48, 49), proliferation (49), cell cycle progression (50), survival (51), and DNA damage (52). Together with current data, this demonstrates the importance of EseN in the modulation of HKDM apoptosis.

Interestingly, monocytes undergoing spontaneous apoptosis *in vitro* changed their cytokine production profile and are characterized by upregulation of IL-10 (23). These differences are seen both at the protein and mRNA levels and directly correlate with the appearance of apoptotic cells in the culture (23). *IL-10* mRNA was also upregulated in HKDM infected with the *∆eseN* mutant at 3 h PI (Fig. 5), indicating that EseN is involved in the inhibition of pro-apoptotic *IL-10* mRNA production during this time.

Caspases play an important role in the initiation and activation of programmed cell death. Caspase-9 triggers intrinsic or mitochondrial signaling pathways of apoptosis, while caspase-8 triggers extrinsic or cell surface receptor pathways (53). Initiation of either of these pathways leads to activation of the executioner caspase-3 and caspase-7, which activate substrates that mediate the changes that characterize apoptotic cells. The significant and early inhibition of extrinsic initiator caspase-8 activity at 1 and 3 h PI in the WT-infected cells compared to the lack of inhibition by *∆eseN* indicates that EseN acts to repress caspase-8 activity. This EseN activity would prevent initiation of apoptosis by infection with WT. The lack of differences between HKDM infected with *E. ictaluri* WT and *ΔeseN* in intrinsic initiator caspase-9 activity (Fig. 6) indicates that EseN is not significantly involved in modulation of caspase-9 activity. Interestingly, executioner caspase-3/7 activity is significantly lower at 3 h post-infection for WT compared to *∆eseN*, presumably because of the suppression of caspase-8 at 1 and 3 h PI by EseN. The WT, however, is not significantly different from *∆eseN* after 5 h post-infection. The increase in caspase-8 at 5 h PI in the WT would account for the WT increase in caspase-3/7, suggesting that the suppressive effect of EseN ends and/or some other factor activates caspase-8 (Fig. 6).

Inflammasome activation is an important innate immune activity that regulates at least two host responses that are protective against infections: (i) secretion of the pro-inflammatory cytokines IL-1β and IL-18 and (ii) induction of pyroptosis, a form of cell death that is triggered by inflammation. Production of IL-1β and IL-18, as well as induction of pyroptosis in infected cells, is protective against many infectious agents (54). Activation of inflammasomes by *Yersinia pestis* depends on the T3SS early in the infection, but later, it is antagonized by the T3SS effector YopK (55, 56). Infection of HKDM with both WT and Δ*eseN E. ictaluri* induces production of pro-IL-1β, but maturation of pro-IL-1β into IL-1β is inhibited. Infection with the total T3SS mutant 65ST, which does not induce any of the T3SS effectors, allowed maturation of pro-IL-1β into Il-1β, indicating that *E. ictaluri T3SS* effectors other than EseN are involved in the suppression of this response (Fig. 8).

In summary, EseN is required for efficient replication of *E. ictaluri* in catfish HKDM and for maximum virulence in the catfish host (16), and our data help to explain this process. Specifically, our data demonstrate that EseN inactivates p38 and JNK MAPKs that play an immunosuppressive role and can lead to fish mortality. EseN also is involved in inhibition of apoptosis and prolongs HKDM survival, which prolongs *E. ictaluri* replication in infected HKDM. Also, for the first time, we demonstrated that EseN is involved in the inactivation of PDK1. However, the role EseN plays in PDK1 inactivation and the biological consequences remain to be studied.

## MATERIALS AND METHODS

### Bacterial strains

*Edwardsiella ictaluri* WT strain 93–146, T3SS knockout mutant 65ST (2), and T3SS EseN effector mutant ∆*eseN* (34) were grown for 16–18 h at 28°C to an OD_600_ = 1.8 to 2.0 in porcine brain-heart infusion broth. All strains grown in broth were aerated on a Max Q4450 incubated shaker (Thermo Scientific, Marietta, OH, USA).

### Infection procedure

Isolation of HKDM was performed as previously described (57). The HKDM cells were seeded in six-well plates for Western blotting, RNA isolation, and TEM, and in 24-well plates for caspase activity. For *E. ictaluri* infection, respective bacterial strains were opsonized for 30 min in normal autologous serum and added in duplicate (for RNA purification, cell lysate collection, and electron microscopy [EM]) or triplicate (for caspase activity and LDH release) wells with HKDM cultures at a multiplicity of infection (MOI) of 10 bacteria to 1 HKDM. Uninfected cells were used as a negative control. After infection, plates were centrifuged at 500 × *g* for 5 min to synchronize contact of the bacteria with the adhered cell layer and were allowed to incubate for 30 min, after which 100 µg/mL gentamicin was added for 1 h at 28°C to kill any remaining extracellular bacteria. Finally, wells were washed once with channel catfish RPMI (ctRPMI) (57), after which channel catfish macrophage media (57) containing a 1-µg/mL bacteriostatic dose of gentamicin was used to control the extracellular growth of any bacteria released from the cells during the experiment.

### *Ex vivo* dephosphorylation assay

To evaluate subsequent dephosphorylation and inactivation of p38, JNK, and PDK1 by EseN, HKDMs were harvested and infected with WT, ΔEseN, or the ΔeseN/eseN complemented strains of *E. ictaluri*. The p38 and JNK pathways were activated after 5 h with the addition of 1 µg/mL of PMA or 20-µM anisomycin for 15 min. PMA is a specific activator of group A (α, βI, βII, and γ) and group B (δ, ε, η, and θ) protein kinase Cs that lead to transient activation of ERK1/2, p38, and JNK (58), while anisomycin is a specific activator of p38 and JNK (24). PMA also activates PI3K (25), which can lead to PDK1 activation. Uninfected, PMA/anycomycin-untreated cells served as negative controls, while uninfected, PMA/anisomycin-treated cells served as positive controls. After PMA/anisomycin treatment, cell lysates were collected in radioimmunoprecipitation assay (RIPA) buffer (Cell Signaling Technology) supplemented with phenylmethylsulfonyl fluoride (PMSF) (Thermo Scientific), Halt Protease Inhibitor Cocktail (Thermo Scientific), and PhosSTOP (Roche Diagnostic GmbH). Before loading into the gel, samples were mixed with 4× protein loading buffer (LI-COR, Lincoln, NE, USA) supplemented with 2-mercaptoethanol (Sigma-Aldrich, St. Louis, MO, USA), boiled for 5 min, separated on a PAGE gel, and transferred to a polyvinylidene difluoride membrane (PVDF), blocked with 2.5% skim milk. Blots were visualized with rabbit anti-pp38, anti-PJNK, or pPDK1 (Ser241) antibody (Cell Signaling Technology). Anti-α/β tubulin was used as a loading control.

### Y2H assay

Y2H assay was performed as previously described (16). The ProQuest Two Hybrid System (Invitrogen, Carlsbad, CA, USA) was employed to identify the specific protein binding partner for T3SS effector EseN. The HKDM cDNA library was cloned into pDEST22 to serve as the prey. EseN fused in frame to the GAL4 BD was constructed on pDEST32 to serve as the bait (16). The bait and prey plasmids were then transformed into *Saccharomyces cerevisiae* strain MaV203 and cultured on YPAD medium. Interaction between prey and bait was detected according to the manufacturer’s instruction.

### RT-qPCR

For RT-qPCR, total RNA extractions were carried out on HKDM samples using RNAzol RT Isolation Reagent (Molecular Research Center, Cincinnati, OH, USA) in combination with the Pure Link RNA mini-kit (Invitrogen) that was used for DNAse treatment and washing steps only, following manufacturer protocols. Samples were resuspended in molecular-grade water (Ambion, TX, USA) and stored at −80°C until use. The RNA concentration and purity were determined using Nanodrop (BioTek Synergy LX Multi-Mode Reader, Daytona Beach, FL, USA) with software Gen5 version 3.11.

The qPCRs were carried out by qPCRBIO SyGreen 1-Step Go Lo-R kit (PCRBiosystems, Wayne, PA, USA). One-step qPCR was performed using 10 ng of respective RNA, 0.5 µM of each gene-specific primer (Table 1) in each reaction mixture under conditions of 54°C for 10 min, 95°C for 2 min, and 40 cycles of 95°C for 5 s and 61°C for 30 s in a LightCycler 96 System (Roche Applied Science, Indianapolis, IN, USA). Oligonucleotide primers were purchased from Integrated DNA Technologies (Coralville, IA, USA). The CanX was used as a reference gene.

**TABLE 1:** Primers used in this work for RT-qPCR

Primers	Forward (5′−3′)	Reverse (5′−3′)
CanX	GCT GTT AAA CCG GAG GAC TG	GCA GGT CCT CGA AGT AGT CAG
apaf1	ACA TCG GCA TCC TGT ACG TC	GCC AGA AAC AGA TCG AAC GC
p53	AGA CAG CCA GGA GTT TGC AG	AGT CCG GGG TAA TCG GAG GT
BCL2	CGG CGG GAT CGT AAG AAG AT	TGA AAA CTG TCT GTC GCG GA
Baxa	TCT GCG ACC CCA CCC ATA AA	CCA CCA CTC TGC CCC AGT TA
IL-10	CTC CTC CCC CTG AGG ATT CA	CGG ATC ACG GCG TAT GAA GA

### Caspase activity

Cultures of HKDM infected with either wild-type *E. ictaluri* or *∆eseN* were inoculated into three replicate wells (for each treatment) of a 24-well plate at an MOI of 10 bacteria per cell. Staurosporine (Sigma-Aldrich)-treated HKDMs (1 µM) were used as a positive control, and untreated HKDMs were used as a negative control. Caspase-8 and caspase-9 activity were measured using the Caspase-8/Caspase-9 Apoptosis Assay Kit (Cell Meter, Sunnyvale, CA, USA). Caspase-3/7 activity was measured using the Apo-ONE homogeneous caspase-3/7 assay (Promega, Madison, WI, USA). Cells in three wells of each treatment were lysed at 1, 3, and 5 h following caspase assay. Fluorescence was measured on a Spectra Max M2 microplate reader (Molecular Devices, Sunnyvale, CA, USA). Caspase activity was expressed as the ratio of infected to uninfected control cells (59).

### Assessment of apoptosis by flow cytometry

Apoptosis was detected in HKDM infected with WT and ∆EseN strains of *E. ictaluri* using Alexa Fluor 647-labeled AnnV and PrI (Thermo Fisher, Waltham, MA, USA) according to the manufacturer’s instructions. Uninfected HKDM and HKDM treated with 1-µM staurosporine were used as controls. After 5 h of infection, macrophages were washed with PBS and removed from the six-well plate in 100 µL of AnnV binding buffer. The cell suspension was then incubated with 5-µL AnnV and 5 µL of PI in the dark for 15 min at room temperature. Finally, 400 µL of binding buffer was added and the samples were analyzed within 1 h on a BD FACSCalibur cell analyzer (Becton, Dickinson and Company, Franklin Lakes, NJ, USA). A total of 50,000 cells were counted in each of the three replicated experiments. Samples were gated on the basis of forward versus side scatter for size, and the results are presented as the percentage of cells that were viable (AnnV−/PrI−), early apoptotic (AnnV+/PrI−), or late apoptotic/necrotic (AnnV+/PrI+).

### Anti-IL-1β antibody preparation

The channel catfish IL-1β gene was amplified from cDNA using a forward primer with sequence added to insert an *NcoI* site just upstream for cloning: CATGccatggATGGCTGACGATTGTTAATGCTGAAA. The reverse primer contains an *XhoI* site: CCGGctcgagATGGCTGACAAAGATTTGTTAATGCTG. The amplified fragment was cloned into the expression vector pET-26b and transferred into Nova Blue competent cells. Constructs were confirmed by plasmid isolation and DNA sequencing and subcloned into B21(DE3), and expression was induced with IPTG. Protein was purified using a HisPur Cobalt purification kit (Thermo Scientific).

The IL-1β antibody was produced in a goat by injecting 1 mL of 1-mg/mL purified IL-1β emulsified in Freund’s complete adjuvant into each hind quadriceps five times at 2-week intervals. The titer of anti-EseN in the serum was determined by dot blot ELISA using purified IL-1β. Specificity of anti-IL-1β antibody was confirmed by Western blotting of whole serum and purified IL-1β.

### Immunoblotting

For immunoblotting, HKDMs were infected with WT, ∆EseN, or T3SS mutant for 5 h as described above. Medium was discarded and lysates from each of two replicate wells were collected using a cell scraper in 50 µL of RIPA cell lysis buffer (Cell Signaling Technology) supplemented with PMSF (Thermo Scientific), Halt Protease Inhibitor Cocktail (Thermo Scientific), and PhosSTOP (Roche Diagnostic GmbH). Immediately after collection, lysates were added with 2× Laemmli Sample Buffer (Bio-Rad) supplemented with 2-mercaptoethanol (Sigma-Aldrich), aliquoted and stored at −80°C until use. Before loading in gel, samples were boiled for 5 min, separated by SDS-PAGE on 12% polyacrylamide gels (Bio-Rad) and transferred onto PVDF membranes (Bio-Rad). Membranes were blocked with 2% bovine serum albumin (Sigma-Aldrich) in Tris-buffered saline (Bio-Rad) with 0.2% Tween 20 (Sigma-Aldrich) for 1 h. The IL-1β protein was detected with IL-1β goat polyclonal antibody (this work). As a loading control, α/β-tubulin was detected using rabbit α/β-tubulin antibody (Cell Signaling Technology). Goat anti-rabbit IRDye 680RD and Donkey anti-goat IRDye 680RD (Li-COR) were used as secondary antibodies, followed by detection on a ChemiDoc MP imaging System (Bio-Rad).

### TEM

The HKDMs were infected for 5 h as described above. Cells were washed with PBS and removed with a cell scraper. After centrifugation, at 500 × *g*, cells were fixed in 2% paraformaldehyde and 1.25% glutaraldehyde in 0.1-M cacodylate buffer with 5% sucrose for 1 to 2 h. The pellet was embedded in 3% agarose, cut into small cubes, washed in buffer, and post-fixed in 1% osmium tetroxide for 30 min–1 h. After rinsing in water, the samples were incubated in 2% uranyl acetate in sodium acetate buffer (pH 3.5) for 2 h, washed in water, dehydrated in a graded series of ethanol/propylene oxide, and embedded in epon-araldite. Thin sections were stained with uranyl acetate and lead citrate and examined in a JEOL JEM 1011 microscope. Samples for TEM were prepared and visualized at the Louisiana State University Electron Microscopy facility.

### Cytotoxicity assay

Leakage of LDH from the cell cytoplasm as a measure of cytotoxicity was quantified using the CytoTox-ONE homogeneous cytotoxicity assay (Promega) at 3 and 5 h PI. A spontaneous release control consisted of supernatant from non-infected HKDM. The maximum release control was the supernatant from cells lysed with 1% Triton X-100 for 1 min. Percentage cytotoxicity was calculated using the following formula: 100% × [(experimental release − spontaneous release)/(maximum release − spontaneous release)], as per the manufacturer’s protocol.

### Statistical analysis

All experiments were performed as three to five independent experiments. Statistical analysis of the data for the p38, JNK, and PDK1 inactivation was conducted by using one-way ANOVA followed by Tukey’s post hoc test for comparison among treatments.

Each RT-qPCR assay was performed in technical triplicates, and relative expression was calculated by the normalized ratio obtained using LightCycler 96 Application Software. To improve this normalization step, CanX was chosen as reference gene. Results were further analyzed by using means from technical replicates of each sample. The relative ratio was calculated for each sample to detect diferences between HKDM-WT and HKDM-EseN. Comparison between two groups was also analyzed by *t*-test. All statistical computations were performed with GraphPad Prism version 5.02 software (GraphPad Software).
